# Evidence Supporting Enrichment of Dissolved Air Gases near Hydrophobic Macromolecules in Aqueous Solutions

**DOI:** 10.1021/acs.langmuir.4c03501

**Published:** 2024-12-30

**Authors:** Ya-Ling Chiang, Yu-Jen Chang, Yun-Ru Chen, Ing-Shouh Hwang

**Affiliations:** † Institute of Physics, Academia Sinica, Nankang, Taipei 115, Taiwan; ‡ Genomics Research Center, Academia Sinica, Taipei 115, Taiwan

## Abstract

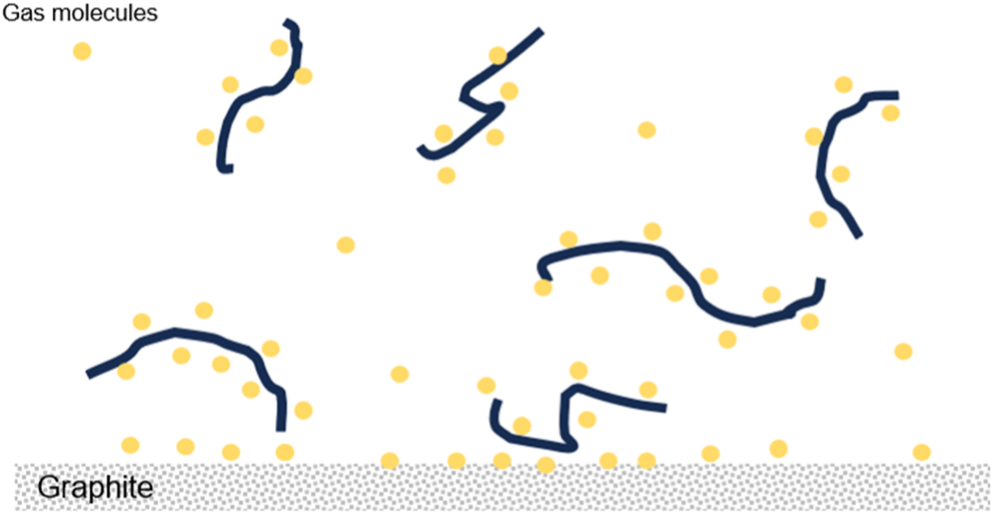

In the present study, we deposited buffer solutions containing hydrophobic (GA)_15_ fibrils onto highly oriented pyrolytic graphite (HOPG) and imaged the surfaces through atomic force microscopy (AFM). Within 3 h of applying ambient (nondegassed) buffers, we observed the formation of two-dimensional stripe-like domains on the HOPG surfaces surrounding the (GA)_15_ fibrils. However, these stripe domains did not form under degassed buffers. Furthermore, the formation of stripe domains on HOPG occurred considerably faster in the presence of buffer solutions containing (GA)_15_ fibrils than in the presence of pure buffer solutions. These stripe structures have recently been identified as nitrogen gas hydrate layers, which are formed through the self-assembly of nitrogen and water molecules on HOPG surfaces. Our findings suggest that gas enrichment occurs in the interfacial water surrounding hydrophobic macromolecules. The results have crucial implications for understanding hydrophobic interactions and the self-assembly of hydrophobic nano-objects in aqueous environments.

## Introduction

Interfaces between water and solid surfaces are crucial to numerous physical, chemical, and biological processes. Although the interfaces between water and hydrophobic materials have been extensively studied for decades, the understanding of interfacial water near hydrophobic surfaces remains incomplete. The effect of dissolved air gases in water has been overlooked by most experimental and theoretical studies, because of their considerably low concentrations under ambient conditions (approximately 10 ppm for nitrogen [N_2_] and 5 ppm for oxygen [O_2_] at saturation). However, several studies have indicated that even at these low concentrations, dissolved gases can affect interfacial properties, including the stability of colloids^1^ and emulsions,^2^ range of hydrophobic attraction,^3^ efficiency of flotation,^4^ and condition of boundary slip in flowing water.^5^ Molecular dynamics simulations of Lennard–Jones systems demonstrated that gas enrichment near hydrophobic surfaces can exceed bulk liquid gas density by more than 2 orders of magnitude.^6^ In the past two decades, various gas-containing structures, such as cap-shaped surface nanobubbles, two-dimensional (2D) micropancakes, and 2D stripe domains, have been observed on hydrophobic surfaces in water.^7−19^ These structures are considered to be the evidence of gas enrichment on hydrophobic surfaces. Gas enrichment on hydrophobic surfaces has been proposed as a key factor in promoting gas hydrate formation by hydrophobic solid particles.^20^ In addition, high-sensitivity atomic force microscopy (AFM) measurements have revealed that the hydration spacing near hydrophobic flat surfaces (0.4–0.6 nm) is substantially larger than that near hydrophilic solid surfaces (0.2–0.3 nm) in ambient water or aqueous solutions.^21−24^ Furthermore, when a degassed solution was used, normal hydration spacing (0.3 nm) was observed on highly ordered pyrolytic graphite (HOPG), a mildly hydrophobic substrate.^19^ These AFM studies suggest that dissolved air gas enrichment occurs within approximately 1.5 nm of hydrophobic flat solid surfaces. The aforementioned findings have been mainly observed on flat, extended hydrophobic solid surfaces in water. However, whether similar gas enrichment occurs in the interfacial water surrounding hydrophobic nano-objects or macromolecules remains unclear. Such gas enrichment may affect interactions among hydrophobic nano-objects, colloids, or macromolecules suspended in water, thereby affecting their self-assembly behavior.

In the present study, we deposited buffer solutions containing hydrophobic (GA)_15_ fibrils onto HOPG and studied the surfaces through AFM. Our previous study demonstrated that after incubation in ambient phosphate buffer, (GA)_15_ peptides rapidly form amyloid fibrils with a cross-β structure.^25^ In another study, we diluted (GA)_15_ fibril solutions with buffers of varying gas conditions and deposited these solutions onto HOPG. This approach resulted in the aggregation of fibrils into morphologies that differed in accordance with gas conditions.^26^ In the present study, within 3 h of deposition in ambient (nondegassed) buffers, we observed the formation of 2D stripe domains (row-like structures) on HOPG outside the (GA)_15_ fibrils. These stripe domains did not form when degassed buffers were used. Similar stripe domains have been reported in several AFM studies of HOPG surfaces in ambient pure water, aqueous solutions, or pure water supersaturated with air gases, particularly N_2_.^14−16,18,19,27−29^ A recent study has identified these stripe structures as nitrogen gas hydrate layers formed by the self-assembly of N_2_ and water molecules on HOPG surfaces.^30^ The considerably faster formation of stripe domains on HOPG in buffer solutions containing (GA)_15_ fibrils, compared to pure buffer solutions, suggests the enrichment of dissolved air gas around hydrophobic macromolecules.

## Experimental Section

### Peptide Synthesis and Preparation

(GA)_15_ is a 30-residue peptide composed of 15 repeats of glycine–alanine dipeptides; both these amino acids are hydrophobic. (GA)_15_ was produced through solid-phase peptide synthesis at the Genomics Research Center, Academia Sinica, Taiwan. To eliminate preaggregates, 4 mg/mL of the (GA)_15_ peptide was dissolved in hexafluoroisopropanol (HFIP, Sigma) and incubated at room temperature for 2 h. After incubation, HFIP was evaporated under vacuum, and the peptide was subsequently dissolved in dichloroacetic acid (DCA, Sigma). The (GA)_15_ solution in DCA was then added to 100 mM sodium dihydrogen phosphate (Na_2_HPO_4_) buffer, and the pH was adjusted to 7.4, resulting in a final DCA concentration of 0.1%. The peptide suspension (at a concentration of 100 μM) was stored at room temperature for several hours, resulting in fibril formation.^25^ Before AFM imaging, the fibril solution was vortexed and gently diluted to the desired concentration by using either degassed or ambient buffer, and the solution was then applied to a freshly cleaved HOPG substrate.

### Preparation of Ambient and Degassed Buffers

To prepare the ambient buffer solution, the pH of 100 mM Na_2_HPO_4_ buffer was adjusted to 7.4, and 0.1% DCA was added. For the degassed buffer, multiple tubes containing 2 mL of ambient buffer were placed in a desiccator and subjected to a vacuum of approximately 0.1 atm by using an oil-free vacuum pump (Rocker 410, Rocker). The buffer was stored in the desiccator for >24 h before use. Before each experiment, the O_2_ concentration of the buffer was measured by using an O_2_ meter (PCD650, Eutech). The results revealed that the O_2_ concentration was approximately 6.5 mg/L for ambient buffer and 1.6 mg/L for degassed buffer. All procedures were conducted at room temperature.

### AFM

AFM measurements were performed using a Bruker AXS Multimode NanoScope V equipped with a commercial fluid cell tip holder. Before and after each experiment, the fluid cell was cleaned by immersing it in ethanol and subjecting it to sonication for at least 30 min, followed by rinsing with DI water. The substrate for sample adsorption was a square piece of HOPG with the lateral dimensions of 12 × 12 mm^2^ (ZYH; Structure Probe, Inc.). HOPG was glued to a round metal chip that was magnetically fixed to the AFM stage. The HOPG sample was freshly cleaved with scotch tape prior to each AFM experiment. The peptide-containing solution was injected onto the HOPG substrate in the liquid cell. The surface was then imaged using the peak force tapping mode in a liquid environment. For imaging, backside gold (Au)-coated silicon (Si) cantilevers (Nanosensors, FM-AuD) were used, with a resonance frequency of 22–32 kHz in the buffer solution and a spring constant of 2–4 N/m. The nominal tip radius was approximately 10 nm. Prior to measurements, the AFM probe was cleaned with ultraviolet (UV) light (UVP CL-1000 UV CrossLinker) for approximately 10 min, making the tip more hydrophilic. During imaging, the scan rate was set to approximately 1 Hz, and the peak force was adjusted to 125–550 pN. All AFM imaging procedures were performed at room temperature in a liquid environment by using the fluid cell tip holder.

## Results and Discussion

### Deposition of (GA)_15_ Fibrils on HOPG in Phosphate Buffer

**Figure 1:**
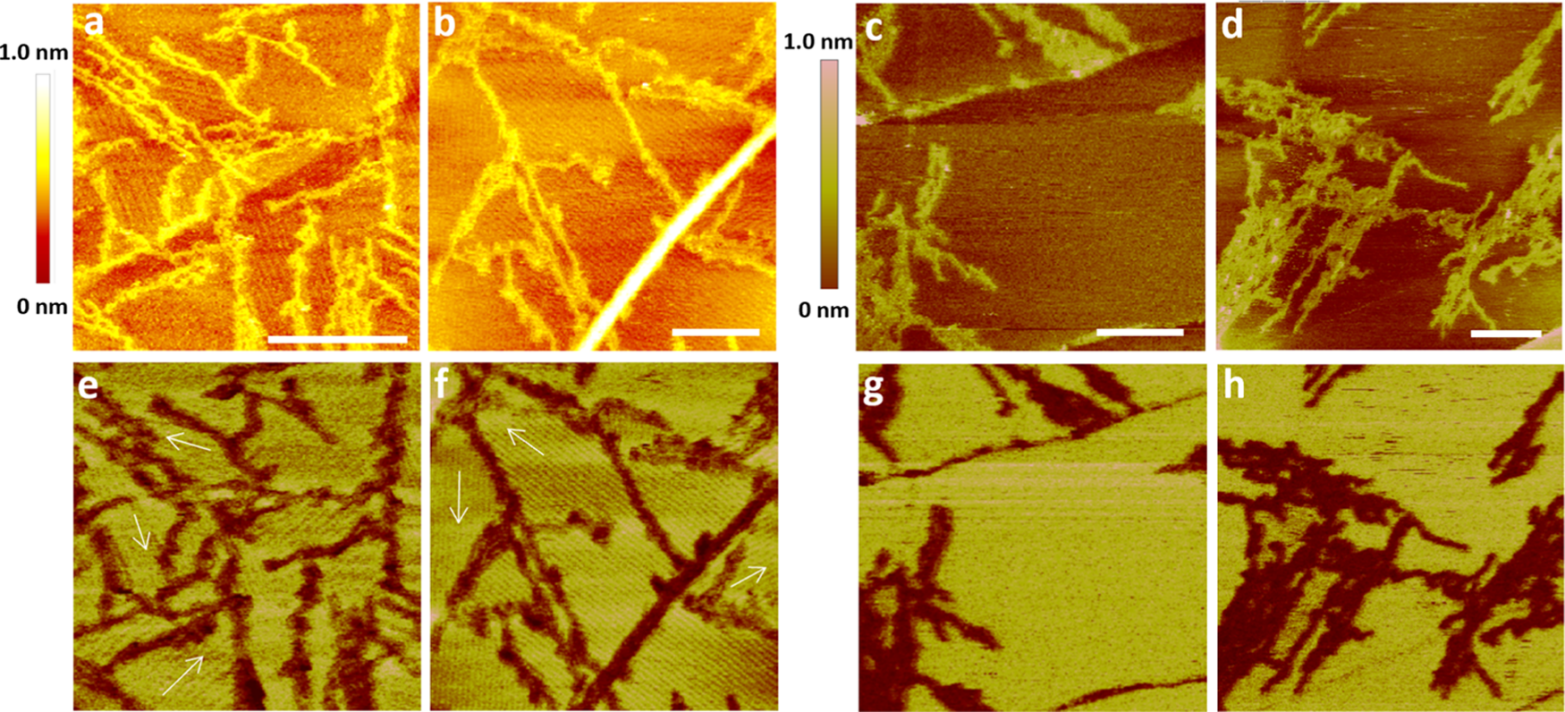
Presence of stripe domains on HOPG surfaces surrounding the (GA)_15_ fibrils under ambient buffer conditions. (a, b) Height image acquired after the deposition of ambient buffer with the (GA)_15_ concentrations of 5 and 2 μM, respectively. The images were acquired at 76 and 105 min, respectively. Time points are relative to the time of buffer deposition (*t* = 0). (c, d) Height image of (GA)_15_ fibrils acquired at 16 and 71 min in degassed buffer, respectively. The concentration of (GA)_15_ was 2 μM. (e–h) Corresponding adhesion maps acquired along with (a–d), respectively. White arrows indicate the stripe orientation. Three orientations reflect the 3-fold symmetry of the HOPG substrate. Scale bar = 100 nm.

The (GA)_15_ peptide, composed of glycine and alanine, is a hydrophobic molecule because both these amino acids lack any charge. (GA)_15_ peptides self-assemble into fibrils in ambient buffer.^25^ Given that HOPG is mildly hydrophobic, in aqueous solutions, these hydrophobic fibrils are readily adsorbed onto the HOPG surface. In this study, after the dilution of the (GA)_15_ fibril solution with either ambient or degassed buffer, the solution was immediately deposited onto a freshly cleaved HOPG substrate. Our AFM imaging revealed that the (GA)_15_ fibrils exhibited a bright contrast in height maps and a dark contrast in adhesion maps (Figure 1). This finding is consistent with that of our previous study.^26^ For the solution diluted with ambient buffer, high-resolution images showed stripe domains covering substantial portion of the HOPG surface outside the fibrils. Figure 1a,b present height images obtained at (GA)_15_ concentrations of 5 and 2 μM, respectively. Figure 1e,f are the corresponding adhesion maps for Figure 1a,b, respectively. The HOPG surface was largely covered by stripe domains, which were visible in the height maps and more pronounced in the adhesion maps. The stripe spacing was 4–6 nm.

We conducted seven similar AFM experiments. In four of them, we achieved stable AFM imaging and the stripe structures were observed in all of these. In the other three experiments, we encountered instability in AFM imaging, likely due to strong hydrophobic attraction when both the tip surface and the sample are hydrophobic. Our AFM tips were typically cleaned with UV treatment right before the experiments, making the tip more hydrophilic. However, the chance of preparing a completely hydrophilic tip through this method is very low (<1% based on our experience). There is always a certain degree of hydrophobicity on the tip surface.^31^ Stable AFM imaging can be achieved if the degree of hydrophobicity on the tip surface is not high. Additionally, the (GA)_15_ molecules are hydrophobic, and there is a chance that the molecules are adsorbed onto the tip.

By contrast, when a (GA)_15_ fibril solution diluted with degassed buffer was deposited on HOPG, no stripe domains were observed outside the fibril regions within the first 2 h (Figure 1c,d,g,h). Two experiments were performed and consistent results were obtained. This finding indicates that the formation of the stripe structures (Figure 1a,b) is related to the air gases dissolved in water. In addition, in the degassed buffer, the fibrils exhibited stronger lateral associations and formed relatively more 2D patches of various shapes compared with those observed in ambient buffer; this finding is consistent with that of a previous study.^26^

**Figure 2:**
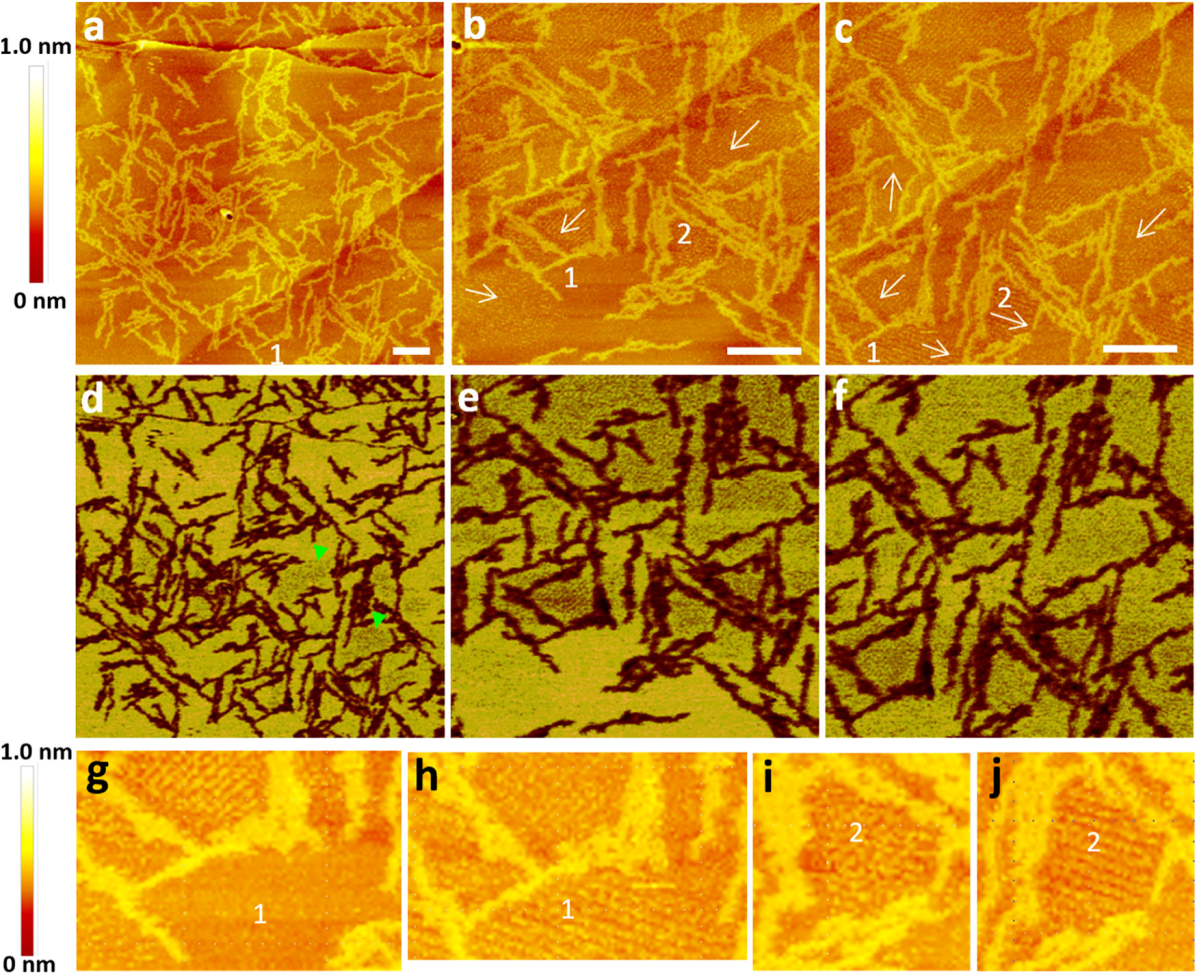
Self-assembly of stripe domains on an HOPG surface surrounding the (GA)_15_ fibrils under ambient buffer conditions. The (GA)_15_ concentration was 5 μM. (a–c) Height images acquired at 57, 59, and 66 min, respectively. (d–f) Corresponding adhesion images for (a–c), respectively. White arrows indicate the orientation of stripe domains. Scale bar = 100 nm. (g, h) Enlarged view of a region around position 1 in (b, c), respectively. The contrast of stripe domains is enhanced. (i, j) Enlarged view of a region around position 2 in (b, c), respectively. The contrast of stripe domains is enhanced. Because of thermal drifts and nonlinearity of the AFM piezo-scanner, a certain degree of image distortion, which varied over time, is visible in these three sets of data.

When we began high-resolution AFM imaging at an early time after depositing the ambient buffer containing (GA)_15_ fibrils onto the HOPG surface, we observed the gradual formation of stripe domains over time (Figure 2). Figure 2a–c presents height images acquired at 57, 59, and 66 min, respectively, and Figure 2d–f displays the corresponding adhesion maps. The (GA)_15_ fibrils exhibited a bright contrast in the height image and a dark contrast in the corresponding adhesion map. Although we noted minimal changes in the fibril organization over time, the progressive formation of stripe domains became increasingly evident. In the large-area image acquired at 57 min (Figure 2a,d), the stripe structures were not resolvable at this resolution. However, in higher-resolution images acquired at 59 min (Figure 2b,e), the stripe structures were visible, with white arrows in Figure 2b indicating the orientation of the stripe domains. Each domain was largely confined by the surrounding (GA)_15_ fibrils. The stripe domains appeared darker in the adhesion maps than in the bare HOPG regions. On the basis of this contrast, a few darker regions (two of them are indicated by green arrow heads in the low-resolution adhesion map in Figure 2d) were likely to contain stripe domains. We also observed the formation process of the stripe domains. For example, at position 1, no stripe structure was visible at 59 min (Figure 2b,g), but a stripe domain had formed by 66 min (Figure 2c,h). At position 2, a stripe domain was fluctuating at 59 min (Figure 2b,i), and it became more stable by 66 min (Figure 2c,j).

**Figure 3:**
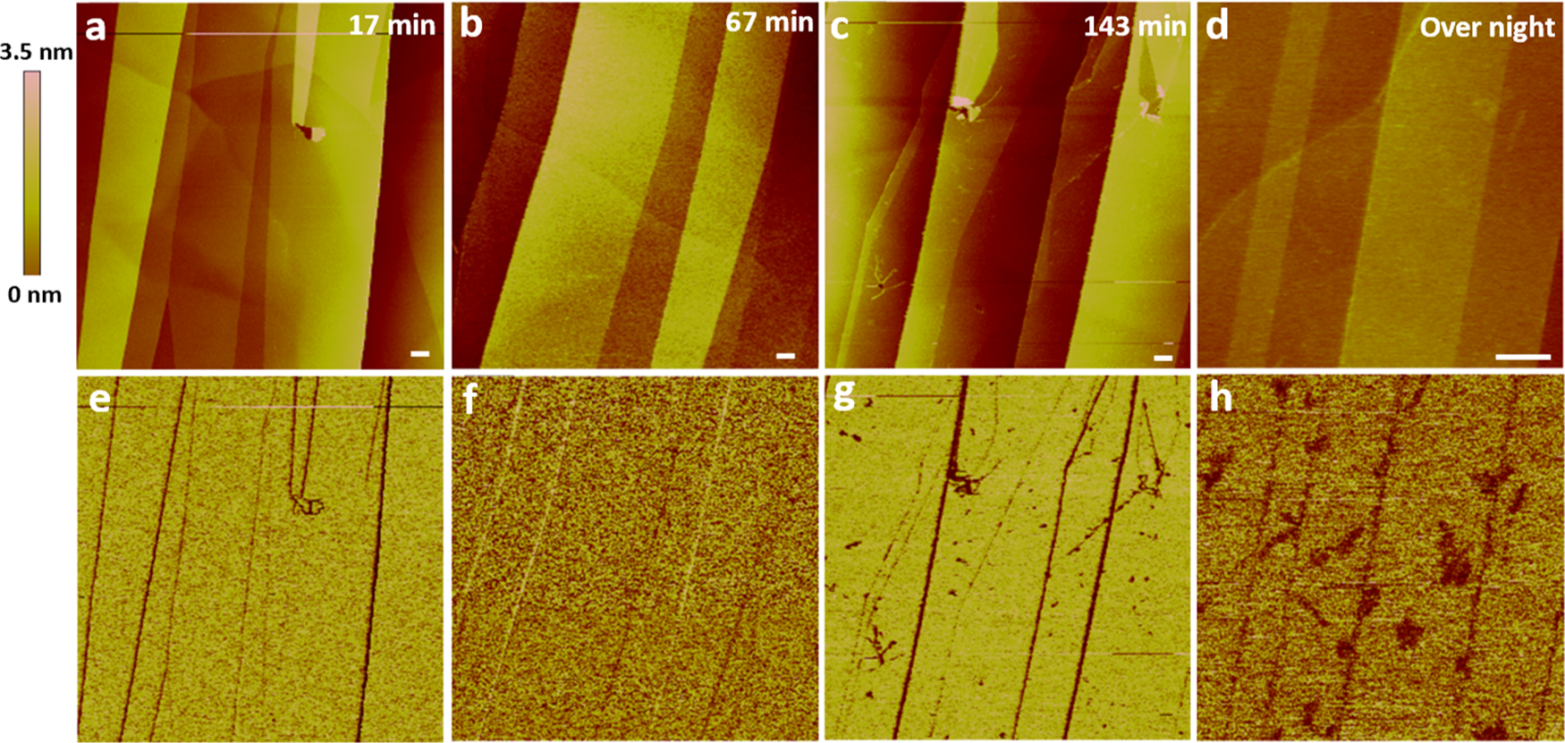
AFM of a HOPG surface under ambient buffer over time. (a–d) Height images acquired at different time points after buffer deposition on HOPG. The time is indicated at the upper-right corner of each set of image. Scale bar = 100 nm. (e–h) The corresponding adhesion map acquired along with (a–d), respectively. The domains of stripe structures (dark spots) are more evident in the adhesion maps. Small domains of the stripe structure are more fragile than those of large domains against AFM scanning.^14,15^ Thus, small domains can be detected only under low-resolution imaging when the stripe pattern is often not resolved.

We also deposited pure ambient buffer without (GA)_15_ onto the HOPG surface and conducted AFM imaging over time. The formation of stripe domains occurred significantly more slowly compared with the deposition of ambient buffer containing (GA)_15_ fibrils (Figure 3). Barely any stripe domains were observed in the height and adhesion maps at 17 min (Figure 3a,e) and 67 min (Figure 3b,f). Very small stripe domains with minimal coverage began to appear at 143 min (Figure 3c,g). After an overnight period, the coverage increased slightly (Figure 3d,h) but still remained below 10%. This finding indicates that the presence of (GA)_15_ fibrils in ambient buffer considerably accelerated the formation of stripe domains on HOPG.

The nucleation and growth of stripe structures (with a height of approximately 0.5 nm and row separations of 4–6 nm) on the freshly cleaved HOPG surface in deionized water and aqueous solutions have been reported previously.^14−16,19^ Similar stripe domains have also been observed on graphene (on mica substrates) in nondegassed deionized water.^18,24^ These studies have suggested that the stripe structures are formed through the rearrangement of water and dissolved air gases, particularly N_2_ molecules, at the HOPG–water interfaces. Combining AFM, X-ray photoemission spectroscopy, and thermal desorption spectroscopy, a more recent study identified these stripe structures as interfacial N_2_ gas hydrate overlayers composed of approximately 90% water and 10% N_2_ molecules.^30^ The stripe structures observed in the present study resemble those reported in earlier AFM studies on HOPG in pure water. Consistent with these earlier findings, the stripe structures did not form when degassed buffer was used (Figure 1c,d,g,h). Thus, we attribute the stripe structures observed in the present study to interfacial N_2_ gas hydrate overlayers.

There have been reports suggesting that the formation of stripe structures on HOPG is due to the adsorption and self-assembly of midlength normal alkanes (20–26 carbon atoms) present in the environment.^32,33^ In our AFM experiments, we used a closed fluid cell, which was cleaned by immersing it in pure ethanol and subjecting it to sonication for at least 30 min before the experiment. Additionally, our solutions did not contain midlength normal alkanes (20–26 carbon atoms). Furthermore, the concentration of midlength normal alkanes in water is extremely low (below 1 ppb),^34^ making it very unlikely for these alkanes to form stripe structures on freshly cleaved HOPG within a few hours of buffer deposition. Another piece of evidence is that the stripe structures did not form under degassed buffer (Figure 1c,d,g,h).

In the current study, we demonstrated that the addition of (GA)_15_ to the buffer solution considerably accelerated the nucleation and growth of stripe domains (Figures 1–3). In solutions containing (GA)_15_, the coverage of stripe domains exceeded 50% of the surface within 80 min after buffer deposition, which is significantly faster than the coverage observed in pure water or aqueous solutions without biomolecules (less than 30% coverage within 120 min after water deposition).^14−16,30^ This rapid formation is likely due to the enrichment of dissolved air gases, particularly N_2_, in the interfacial water surrounding the hydrophobic macromolecules. When these hydrophobic macromolecules are adsorbed onto HOPG, the surrounding gas molecules (mainly N_2_) come into contact with the HOPG surface, leading to even greater N_2_ enrichment in the interfacial water on the HOPG surface. This gas enrichment facilitates the formation of stripe domains, explaining the observations in the present study.

Chen et al. reported that dissolved gases can substantially affect the aggregation behavior of carbon nanotubes (CNTs) dispersed in water. These CNTs, which are hydrophobic nano-objects, form loose, gel-like structures resembling semidilute polymer solutions in ambient (nondegassed) water, However, when CNTs are mixed with degassed water, they primarily exist as isolated aggregates of submillimeter size that sediment rapidly.^35^ The study indicates that dissolved air gases prevent CNTs from forming large, dense aggregates that could precipitate quickly.^35^ In addition, the results reveal that even after several cycles of free-pump-thaw, which is a highly effective degassing procedure for pure water, some gas remains when CNTs are dispersed in water. This finding indicates that CNTs retain some dissolved gas after cycles of free-pump-thaw, suggesting a high affinity between dissolved gases and hydrophobic nano-objects in water.^35^ Moreover, the formation of isolated aggregates in degassed water is consistent with the stronger lateral association of (GA)_15_ fibrils observed in degassed buffer solutions.^26^

**Figure 4:**
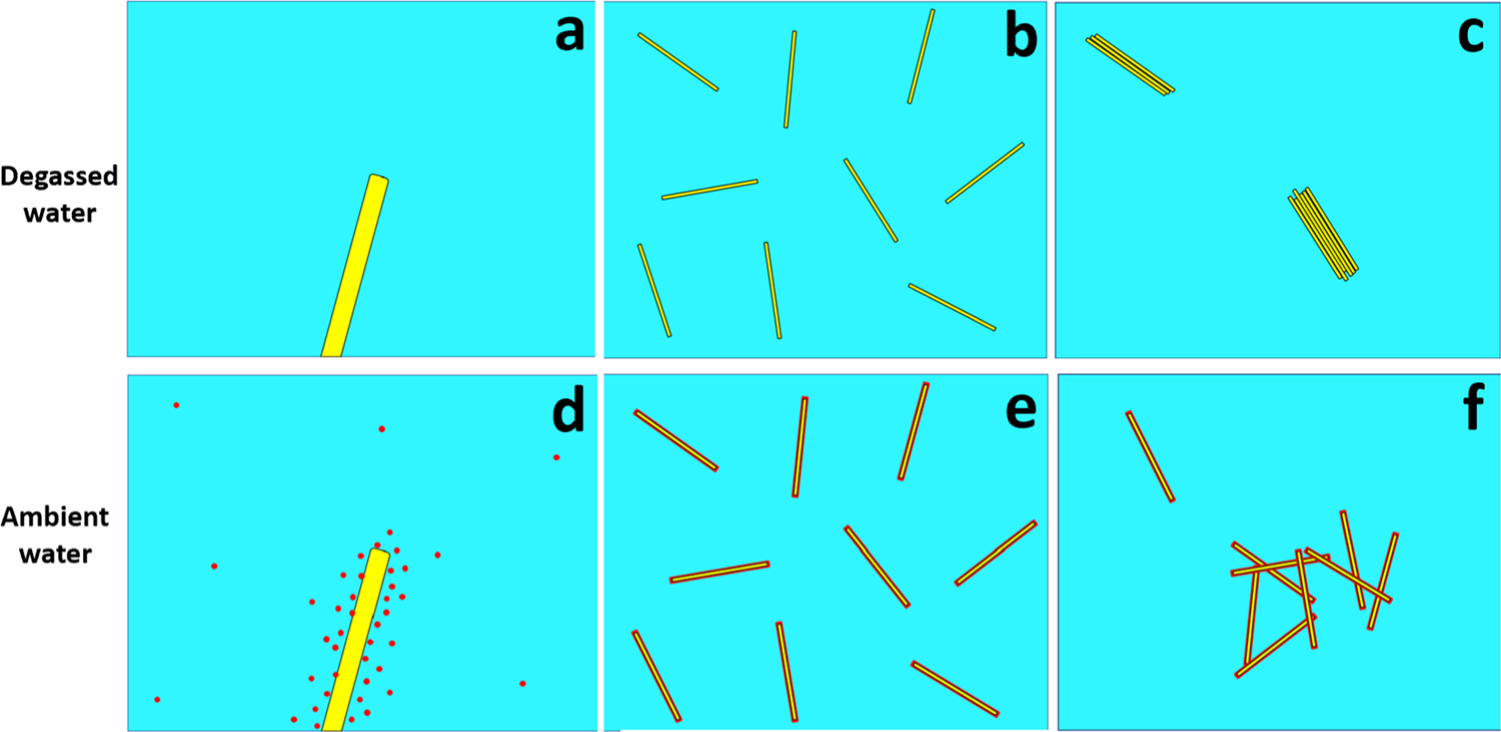
(a) Schematic for an individual hydrophobic nano-object suspended in a degassed aqueous solution. (b) Initial condition with individual hydrophobic nano-objects suspended in a degassed aqueous solution. (c) Aggregation of hydrophobic nano-objects in a degassed aqueous solution. (d) Schematic for an individual hydrophobic nano-object suspended in an ambient (nondegassed) aqueous solution. (e) Initial condition with individual hydrophobic nano-objects suspended in an ambient (nondegassed) aqueous solution. (f) Association of hydrophobic nano-objects in an ambient (nondegassed) aqueous solution.

Here we postulate that the enrichment of dissolved air gases in interfacial water surrounding hydrophobic nano-objects (or macromolecules) has a thermodynamic driving force. In degassed water, the interfacial tension between water and a hydrophobic nano-object (or a macromolecule) is high (Figure 4a), which may lead to stronger hydrophobic interactions among the hydrophobic nano-objects and a stronger aggregation tendency (Figure 4b,c). Under ambient water or aqueous solution, dissolved air gases enrich in the interfacial water surrounding a hydrophobic nano-object to reduces the interfacial tension (Figure 4d). Some gas molecules might adsorb onto the nano-objects; there are also dissolved gas molecules dispersed in the interfacial water next to the nano-objects and the gas concentration is considerably higher than that in the bulk solution (Figure 4d). The gas enrichment may stabilize individual hydrophobic nano-objects (or molecules) suspended in water and the aggregation tendency is reduced (Figure 4e,f). Such a scenario explains the different aggregation behaviors of CNTs in ambient water and degassed water^35^ and the stronger lateral association of (GA)_15_ fibrils observed in degassed buffer solutions.^26^Figure 4 illustrates only one-dimensional (1D) nano-objects, but the general concept can also be applied to nano-objects of other shapes.

In addition to the current and the aforementioned studies, an early experimental and theoretical study demonstrated the presence of N_2_ and O_2_ molecules in normal lipid bilayers prepared without gas control.^36^ The segregation of these dissolved gas molecules within the hydrophobic cores of lipid bilayers affects the properties and stability of the bilayers.^36^ These findings suggest that dissolved air gases play substantial roles in various biological, chemical, and physical systems in water or aqueous solutions under ambient conditions, particularly when there are hydrophobic domains in the systems.

## Conclusions

In the present study, we found evidence supporting the enrichment of gas in the interfacial water surrounding hydrophobic macromolecules. This finding has crucial implications for hydrophobic interactions and the self-assembly of hydrophobic nano-objects in ambient water or aqueous solutions. However, gas enrichment is often overlooked in many theoretical and experimental studies involving hydrophobic domains in ambient aqueous solutions. Additional theoretical and experimental studies should be conducted to investigate the effects of gas enrichment. Such studies can enhance our understanding of numerous phenomena in ambient water or aqueous solutions.
